# Mechanisms of Epstein‐Barr virus nuclear antigen 1 favor Tregs accumulation in nasopharyngeal carcinoma

**DOI:** 10.1002/cam4.3213

**Published:** 2020-06-22

**Authors:** Jie Wang, Yunfan Luo, Pei Bi, Juan Lu, Fan Wang, Xiong Liu, Bao Zhang, Xiangping Li

**Affiliations:** ^1^ Department of Otolaryngology, Head and Neck Surgery Nanfang Hospital Southern Medical University Guangzhou China; ^2^ School of Public Health and Tropical Medicine Southern Medical University Guangzhou China

**Keywords:** Epstein‐Barr virus nuclear antigen 1, M2 macrophage, transforming growth factor β1, Treg

## Abstract

**Background:**

Documented reports proved that Epstein‐Barr virus (EBV) infection increased infiltration of Tregs in malignancy. However, the mechanism of EBV recruitment Tregs into nasopharyngeal carcinoma (NPC) tissues has not been detailed discussion.

**Methods:**

Expression of EBV nuclear antigen 1 (EBNA1) and Foxp3 in NPC tissue samples was detected by immunohistochemistry. EBNA1+ NPC cell lines were used to coculture with PBMC, naïve T cells, Tregs, and monocytes. Percent of Treg was detected by flow cytometry.

**Results:**

EBNA1 protein was overexpressed in NPC tissues, and was associated with a number of infiltrated Tregs. EBNA1+ NPC cells converted naïve T cells into Tregs by up‐regulated TGF‐β1. Enhanced CCL20 production in EBNA1‐expressed tumor cells increased Tregs migration. Polarized‐M2 macrophages by EBNA1 expression cells converted naïve T cells into Tregs.

**Conclusions:**

EBNA1 favors accumulation of Tregs in NPC through: (a) upregulated TGF‐β1 converted naïve T cell into Treg; (b) upregulated CCL20 increased Treg migration; and (c) polarized‐M2 macrophage converted naïve T cell into Treg.

## Graphical abstract

It is well known that Tregs accumulated in several EBV‐associated malignancies. Our previous studies also proved Tregs increased in NPC tissues and peripheral blood. However, the mechanism of EBV recruitment Tregs into NPC tissues has not been discussed in detail. EBNA1 protein was highly expressed in NPC tissue specimens, and its expression was associated with number of infiltrated Tregs. EBNA1 expression NPC cells induced naïve T cells into Tregs by upregulated TGF‐β1. Enhanced CCL20 production in EBNA1‐expressed tumor cell increased Tregs migration. Polarized‐M2 macrophages by EBNA1 expression cells converted naïve T cells into Tregs. These findings provided novel insight into the vital role of EBNA1 in recruitment of Tregs in NPC.

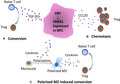

## 1 INTRODUCTION

Epstein‐Barr virus (EBV) is an oncogenic virus that is closely associated with development of several malignancies, including Hodgkin lymphoma (HL), Burkitt lymphoma, nasopharyngeal carcinoma (NPC), and EBV+ gastric carcinoma (GC). EBV‐DNA can be detected in approximately 90% of NPC tissues, where the virus express a limited subset of genes; these include EBV nuclear antigen 1 (EBNA1), latent membrane protein 1 (LMP1), and LMP2. Although massive EBV‐specific cytotoxic T cells (CTLs) can be detected in NPC, they are unable to eliminate EBV‐infected cells in vivo.^1^, ^2^ Furthermore, our previous study found high‐density infiltration of CD8+ T cells in NPC tissues have lower overall survival (OS) and progression‐free survival.^3^ We speculated that is related immune evasion in tumor. Several immune evasion mechanisms have been proposed, one current focus in attempt to understand this phenomenon is regulatory T cells (Tregs).

Within tumor microenvironment, Tregs had been well‐documented recruitment in many different tumor tissues and malignant effusions, and this situation is associated with tumor progression and poor prognostic.^4^ A multicenter, randomized trials showed promise result in early‐phase clinical trials by elimination of Tregs with an anti‐CD25‐coupled toxin.^5^ The clinical benefit of check‐point blockade with anti‐CTLA4 has also been partially attributed to depletion of TI Tregs.^6^ Together, these results suggest that Tregs are promising target for antitumor immunotherapy.^7^

Documented reports proved that EBV infection increased infiltration of Tregs in malignancy. In EBV‐associated GC, the enhanced CCL22 produced by EBV+ tumor cells promoted the accumulation of Tregs.^8^ The migration of Tregs toward tumor significantly increased, and both natural and induced Tregs increased in EBV‐positive HL.^9^, ^10^, ^11^ And further research has shown that EBNA1+ HL cells mediate overexpression of CCL20 which could recruit regulatory T cells migrated into tumor.^12^ There has been a part of literature only demonstrated Tregs increased both in peripheral and tissues of NPC.^13^, ^14^ The relationship of EBV with Tregs in NPC has not been described. Our previous study has proved higher EBV copies have more Tregs in NPC patient peripheral blood.^15^ However, the mechanism of EBV recruitment Tregs into NPC tissues has not been discussed in detail. In this study, we found EBNA1 increased infiltration of Tregs in tumor microenvironment through converting naïve T cell into Tregs via upregulate TGF‐β1, upregulated CCL20 increased Tregs migration, and polarized‐M2 macrophage converted naïve T cells into Tregs.

## 2 MATERIALS AND METHODS

### 2.1 Samples

We collected 177 NPC tissues and 50 nasopharyngitis tissues from Nanfang Hospital of Southern Medical University from 2007 to 2014. The inclusion criterions were as follows: histologically conformed NPC, no previous treatment for NPC, and without other malignancy diseases. The classification of TNM was according to definition of seventh edition of the UICC‐American Joint Committee on Cancer staging criteria. Treatment of patients refers to National Comprehensive Cancer Network (NCCN) for NPC. We performed regular follow‐up for these NPC patients. The median follow‐up was 51 months. The 5‐year OS rate and progression‐free survival rate were 66.8% and 57.6%, respectively. The clinicopathological characteristics were demonstrated in Table 1. This study was approved by the ethics committee of Nanfang Hospital (Southern Medical University, GuangZhou, China).

**TABLE 1:** Clinicopathological characteristics of 177 NPC patients

Clinicopathological characteristics	Number of patients
Gender	
Male/Female	132/45
Median age (y)	45 (38.5‐54)
Median follow‐up time (mo)	51 (31‐60)
Cigarette smoking	
Yes/No	88/89
Alcohol intake	
Yes/No	47/130
Histological subtype	
Undifferentiated nonkeratinizing cacinoma	159
Differentiated nonkeratinizing cacinoma	16
Squamous cell cacinoma	2
T status	
T1/T2/T3/T4	34/33/49/61
N status	
N0/N1/N2/N3	18/68/60/31
M status	
M0/M1	162/15
Clinical stage	
Ⅰ/Ⅱ/Ⅲ/Ⅳ	7/25/59/86
Progress	76
Death	69

### 2.2 Immunohistochemistry

Paraffin‐embedded tissues were sliced as 4‐μm sections. Tissue sections were analyzed with Foxp3 (Abcam), CD163 (Abcam), EBNA1 (Bio‐Rad), TGF‐β1 (R&D System), and CCL20 (R&D System). For counting Foxp3+ Treg and CD163+ M2 macrophage, five high‐power filds (HPF) were randomly selected, the number of positive cells were counted, and the data expressed as the mean of those 5 filds.The other three proteins were as follow. The percent of positive cells: 0; 1%‐24%, 1; 25%‐49%, 2; 50%‐74%, and 3; >75%. Stained intensity scored as: absent, 0; weak, 1; moderate, 2; and strong, 3. Multiply score of positive cells by stained intensity was the final score.^16^

### 2.3 Cell culture and plasmid stable transfection

In our study, human NPC 5‐8F and CNE1 cell lines were cultured. RPMI 1640 medium (Hyclone) with 10% FBS (Gibco) was used to culture these NPC cell lines. To obtained stable overexpression EBNA1 cell line, we transfected PCEP4 (Invitrogen), which containing EBV replication initiation Orip and EBNA1 cDNA into 5‐8F. Hygromycin B was added to selected cells with stable overexpression EBNA1. We also purchased pc‐DNA 3.1 (Invitrogen), which has similar structure as PCEP4 and Hygromycin B resistance, as negative control.

### 2.4 PBMCs, Tregs, and naïve T cells isolation

PBMCs (peripheral blood mononuclear cell) were purified from fresh human blood by gradient centrifugation using Ficoll‐Paque (HY biological manufacture). Magnetic activated cell sorting (MACS) was used to isolate different T‐cell subsets. CD4+ T cells, CD4+CD25+ T cells, and naïve T cells (CD4+CD25− T cells) were isolated by LD (Miltenyi) column and MS column (Miltenyi), respectively.

### 2.5 Conversion and chemotaxis assay of Tregs

Isolated naïve T cells were seeded in six‐well plates with anti‐CD3 (0.5 μg/m:, ebioscience) and anti‐CD28 (2 μg/mL, ebioscience) stimulated for 2 days. Then, cocultured with NPC cells with transwell in the presence or absence of anti‐TGF‐β1 (10 ng/ml, R&D System) for 4 days.

Migration of Tregs was tested by 5 μm pore‐size transwell (Corning Costar). 1 × 10^4^ Tregs were seeded in upper chamber with NPC cells supernatant in lower chamber and cultured with or without anti‐CCL20 (10 ng/mL, R&D System) for 24 hours. The number of migrated cells in the lower chamber was counted by microscope.

### 2.6 Human monocytes isolation

Plastic adherence culture dishes (Corning) were used to culture PBMCs for 4 hours to collect adherent monocytes. Monocytes were cultured in RPMI 1640 medium (Hyclone) with 10% FBS.

### 2.7 Flow cytometry

Cells were stained with anti‐human CD4‐FITC (ebioscience), anti‐human CD25‐APC (ebioscience), anti‐human Foxp3‐PE (ebioscience), and anti‐human CD163‐APC (ebioscience), and analyzed with flow cytometry (Becton, Dickinson and Company).

### 2.8 Animal study

Animal work was approved by the Institutional Review Boards and Animal Care and Use Committees of Southern Medical University. 1 × 10^6^ tumor cells in 200 μL PBS were subcutaneously injected into groin of 6‐ to 8‐week‐old male nude mice. Two weeks later, 1 × 107 PBMC were injected into tail vein. After 48 hours, mice were sacrificed and the isolated tumors were mechanically dispersed. PBMCs were extracted and human Tregs were identified by FACS with an antibody against human CD4 and Foxp3. Tumor growth was evaluated by monitoring tumor volume (TV = length × width^2^ × 0.5) every 2 days.

### 2.9 Statistical analysis

Comparison of two independent groups was made by two‐tailed Student's *t* test. Multiple comparisons among several groups were used for one‐way ANOVA analysis. Spearman correlation analysis was used to analyze the correlation of the two groups. The data are shown as the mean ± SEM unless otherwise noted. Survival analysis was compared by the log‐rank test. The significance of various variables for survival was analyzed by the Cox proportional hazards model. The SPSS 21.0 statistical software package (SPSS) was applied to all statistical analyses. *P* values <.05 were statistically significant.

## 3 RESULTS

### 3.1 Increased Tregs predict poor survival of NPC patients

**FIGURE 1:**
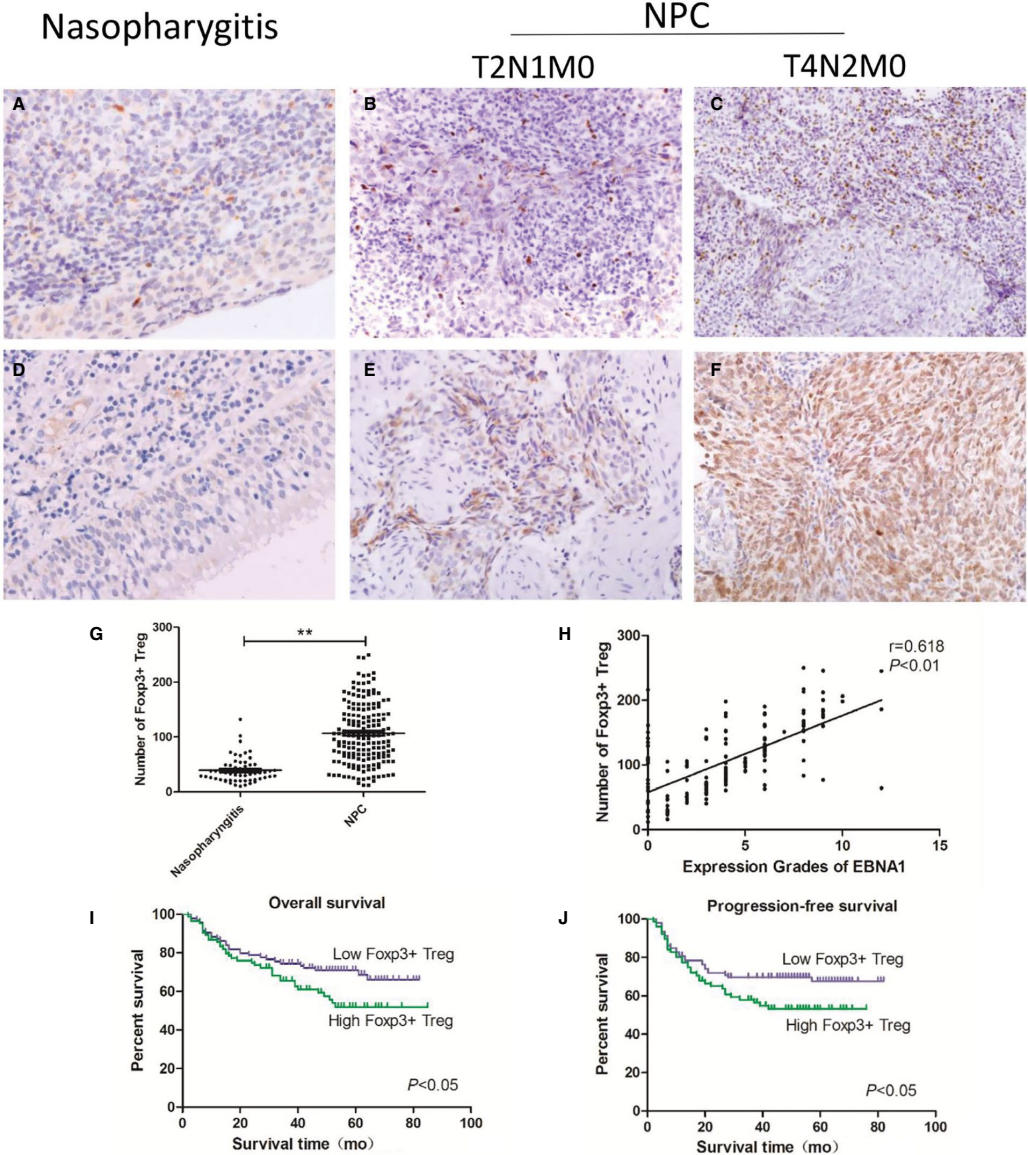
High‐density infiltrated Tregs predict poor survival of NPC patients and had a correlation with EBNA1. (A) Foxp3+ Treg in nasopharyngitis tissue, (B, C) Foxp3+ Treg in different grades of NPC tissue, (D) expression of EBNA1 in nasopharyngitis tissue, (E, F) EBNA1 in different grades of NPC tissue, (G) more Foxp3+ Treg infiltrated in NPC than nasopharyngitis, (H) high‐density infiltrated Tregs had a correlation with EBNA1, and (I, J) high‐density infiltrating Tregs had lower overall survival and lower progression‐free survival

To evaluate the number of Foxp3+ Tregs in NPC, we studied 177 tumor tissues from individuals with untreated patients. Then, chose 50 nasopharyngeal tissues from patients with chronic nasopharyngitis as control. We found a substantial population of Foxp3+ Tregs in NPC tissues (106.7 ± 57.0) (Figure 1B,C) and a small amount in chronic nasopharyngitis (39.3 ± 23.6) (Figure 1A,G).

Accumulation of Foxp3+ Tregs predicts poor survival. Samples were divided into two groups on the basis of the density of tumor infiltrating Tregs. The survival curves indicated that high‐density infiltrating Tregs had lower OS (Figure 1I) and lower progression‐free survival (Figure 1J). We used univariate Cox analysis and analyzed Foxp3+ Tregs and clinicopathological characteristics in NPC patients (Table S1). Multivariate Cox analysis was used to analyze Foxp3+ Tregs and statistically significant clinicopathological characteristics (Table S2). We also analyzed all relevant clinical and pathological information, and found that high‐density infiltrating Tregs have worse clinical stage and metastasis to lymph gland (Table 2).

**TABLE 2:** Association of clinicopathological characteristics and the densities of Foxp3+ Tregs

Clinicopathological characteristics	Foxp3+ Tregs		*P* value
	<106, n = 87	≥106, n = 90	
Gender			
Male	61	71	.18
Female	26	19	
Age			
<45	40	48	.31
≥45	47	42	
Cigarette smoking			
Yes	41	57	.5
No	46	43	
Alcohol intake			
Yes	19	28	.16
No	68	62	
Histological subtype			
Undifferentiated nonkeratinizing cacinoma	76	83	.28
Differentiated nonkeratinizing cacinoma + squamous cell cacinoma	11	7	
T status			
T1 + T2	36	31	.34
T3 + T4	51	59	
N status			
N0 + N1	50	36	**.02**
N2 + N3	37	54	
M status			
M0	80	82	.84
M1	7	8	
Clinical stage			
Ⅰ+Ⅱ	21	11	**.04**
Ⅲ+Ⅳ	66	79	

Note

Bold value: *P* < 0.05.

### 3.2 Expression of EBNA1 has positive correlation with infiltrated Tregs

EBNA1 was detected in 154 of 177 (87.0%) NPC tissues, but was not detected in 50 chronic nasopharyngitis tissues (Figure 1D‐F). Expression of EBNA1 has positive correlation with infiltrated Tregs (Figure 1H).

### 3.3 Constructed EBNA1 expressed NPC cell line

**FIGURE 2:**
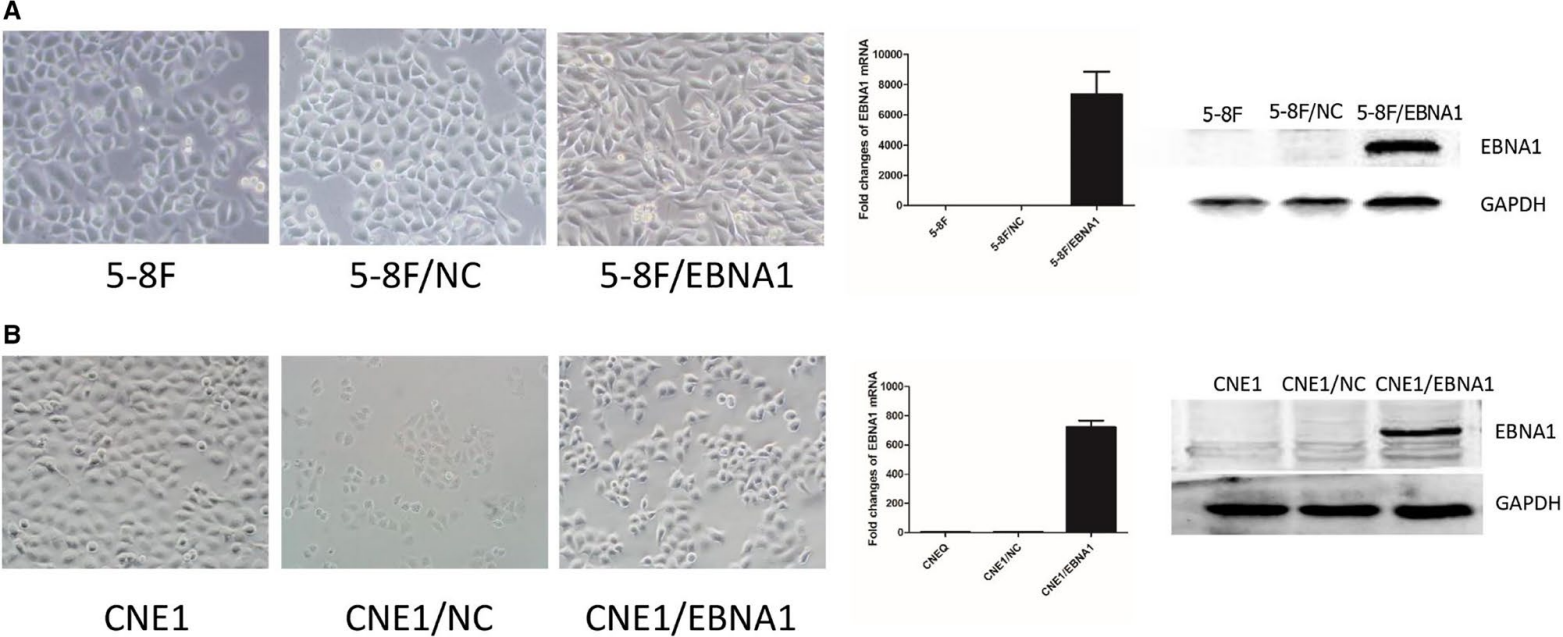
Constructed EBNA1 expressed NPC cell line. A, The morphology of 5‐8F/EBNA1 cell line underwent considerable changes. Western blot and qPCR analysis confirmed that EBNA1 proteins were expressed in 5‐8F/EBNA1. B, The CNE1/EBNA1 has more lamellipodium compared to control. Western blot and qPCR conformed EBNA1 overexpressed

EBNA1 protein was detected in 5‐8F/EBNA1 and CNE1/EBNA1 by Western blot. QPCR analysis also found EBNA1 high expressed in 5‐8F/EBNA1 and CNE1/EBNA1 (Figure 2A,B). The morphology of 5‐8F/EBNA1 cell line underwent considerable changes. The EBNA1+ cell lines became fibroblast like, with a narrow spindle shape and lamellipodiums. But the shape of 5‐8F/NC was not changed compared with 5‐8F. The CNE1/EBNA1 has more lamellipodiums compared with control.

### 3.4 EBNA1‐induced naïve T cells converted into Tregs via TGF‐β1

TGF‐β1 was overexpressed in NPC tissues compared with nasopharyngitis (Figure S1A‐D). TGF‐β1 was mainly expressed in cytoplasm of tumor cells (Figure S1B‐D). TGF‐β1 expression was associated with NPC clinical stage (Figure S1E). Expression of EBNA1 was positively associated with TGF‐β1 expression in NPC (Figure S1F).

**FIGURE 3:**
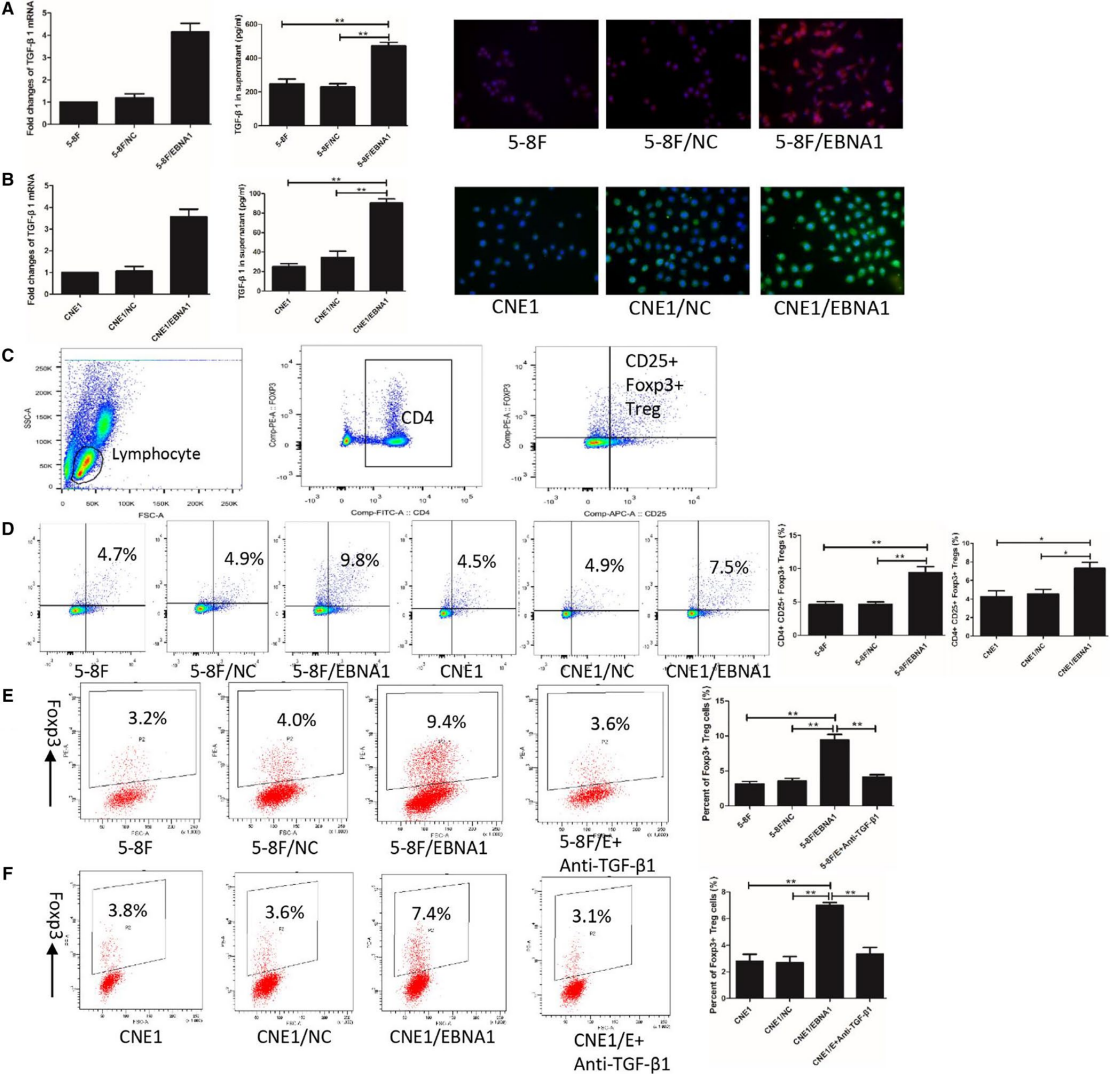
EBNA1 induced naïve T cells converted into Tregs via TGF‐β1. A and B, showed ELISA, qPCR and immunofluorescence proved TGF‐β1 overexpressed in 5‐8F/EBNA1 or CNE1/EBNA1. C, is gate of CD4+CD25+Foxp3+ Tregs. The percentage of CD4+CD25+Foxp3+ Tregs increased after cocultured with 5‐8F/EBNA1 and CNE1/EBNA1 (D). There were more naïve T cells converted into Tregs after cocultured with 5‐8F/EBNA1 or CNE1/EBNA1, and anti‐TGF‐β1 antibody could terminated this phenomenon (EF)

TGF‐β1 was overexpressed in 5‐8F/EBNA1 and CNE1/EBNA1 compared with control. TGF‐β1 mRNA was higher in 5‐8F/EBNA1 and CNE1/EBNA1 (Figure 3A,B). ELISA detected that the supernatant of 5‐8F/EBNA1 and CNE1/EBNA1 had higher TGF‐β1 compared with control (Figure 3A,B). Immunofluorescence also proved that TGF‐β1 was highly expressed in EBNA1‐expressed NPC cell lines (Figure 3A,B).

Nasopharyngeal carcinoma cell lines were cocultured with PBMC for 4 days to explore the interaction of NPC cells and lymphocytes in vitro. Figure 3C showed gating strategy for CD4+CD25+Foxp3+ Treg identification. The percentage of CD4+CD25+Foxp3+ Tregs after cocultured with NPC cells: 5‐8F/EBNA1 (9.47 ± 1.43%), 5‐8F/NC (4.70 ± 0.53%), 5‐8F (4.63 ± 0.70%), CNE1/EBNA1 (7.30 ± 1.11%), CNE1/NC (4.57 ± 0.85%), and CNE1 (4.27 ± 1.07%) (Figure 3D).

To figure out the specific mechanism of increased Tregs, naïve T cells (CD4+CD25− T cells) were isolated with MACS. Naïve T cells cocultured with NPC cells for 4 days. Figure 3E,F showed that Foxp3+ Treg percent was significantly higher in cocultured with 5‐8F/EBNA1 (9.43 ± 1.35%) and CNE1/EBNA1 (7.01 ± 0.38%) compared with cocultured with 5‐8F/NC (3.57 ± 0.67%) or 5‐8F (3.13 ± 0.60%) and 5‐8F/NC (2.70 ± 0.78%) or 5‐8F (2.80 ± 0.89%). Anti‐TGF‐β1 in the coculture system was used to blockade the effects of TGF‐β1. The results revealed that anti‐TGF‐β1 could blockade conversion changes of Tregs in cocultured with EBNA1+ group.

### 3.5 Enhanced CCL20 production by EBNA1 + tumor cells increased Tregs migration

**FIGURE 4:**
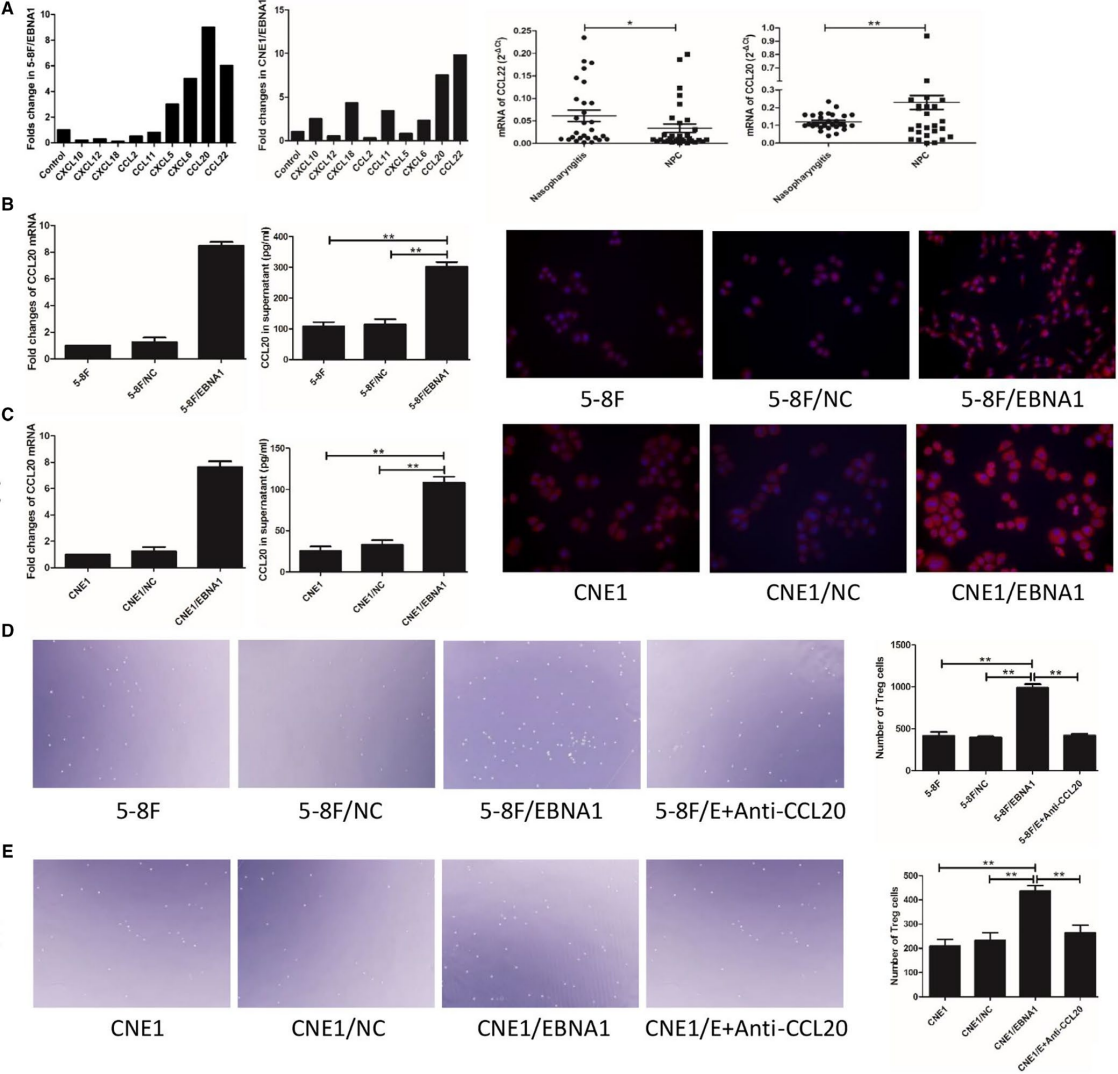
Enhanced CCL20 production by EBNA1+ tumor cells increased Tregs migration. Nine chemokines were detected in EBNA1+ tumor cells, and the results showed CCL20 and CCL22 overexpression (A). CCL20 was overexpressed in NPC tissues (A). CCL20 was overexpressed in EBNA1+ cells (B, C). Supernatant of EBNA1+ cells increased migration of Tregs, and anti‐TGF‐β1 antibody could terminate this condition (D, E)

Nine chemokines were detected in EBNA1+ tumor cells, and the results showed CCL20 and CCL22 overexpressed (Figure 4A). We tested the mRNA of CCL20 and CCL22 in NPC tissues. The results showed CCL20 was overexpressed in NPC tissues (Figure 4A). Expression of CCL20 was mainly found in cytoplasm of tumor cells (Figure S2A). Expression of CCL20 and EBNA1 had a positive correlation in NPC (Figure S2B). Similar to NPC tissues, CCL20 was overexpressed in EBNA1+ cells (Figure 4B,C). Figure 4B,C showed that mRNA of CCL20 was higher in EBNA1+ cells. CCL20 in 5‐8F/EBNA1 (302.33 ± 26.5 pg/mL) supernatant enhanced production compared with 5‐8F (108.36 ± 23.16 pg/mL) and 5‐8F/NC (114.25 ± 29.74 pg/mL) supernatant, and in CNE1/EBNA1 was 108.67 ± 12.16 higher than 5‐8F 25.36 ± 9.61 pg/mL and 5‐8F/NC 33.33 ± 9.07 pg/mL. Immunofluorescence images were consistent with qPCR and ELISA data.

CD4+CD25+ Tregs were isolated with MACS. There were more migrated Tregs when cocultured with supernatant of 5‐8F/EBNA1 (989 ± 74) compared with 5‐8F (413 ± 81) and 5‐8F/NC (396 ± 22) supernatant, and a saturated concentration of monoclonal antibody to CCL20 significantly blocked 5‐8F/EBNA1 supernatant (418 ± 32)‐induced Tregs migration (Figure 4D). In CNE1 group, migrated Tregs in CNE1/EBNA1 supernatant was 437 ± 38, CNE1 supernatant was 210 ± 46, CNE1/NC supernatant was 233 ± 55, and 5‐8F/EBNA1 with antibody to CCL20 supernatant was 263 ± 57 (Figure 4E).

### 3.6 Polarized‐M2 macrophage by EBNA1 converted naïve T cell into Treg

**FIGURE 5:**
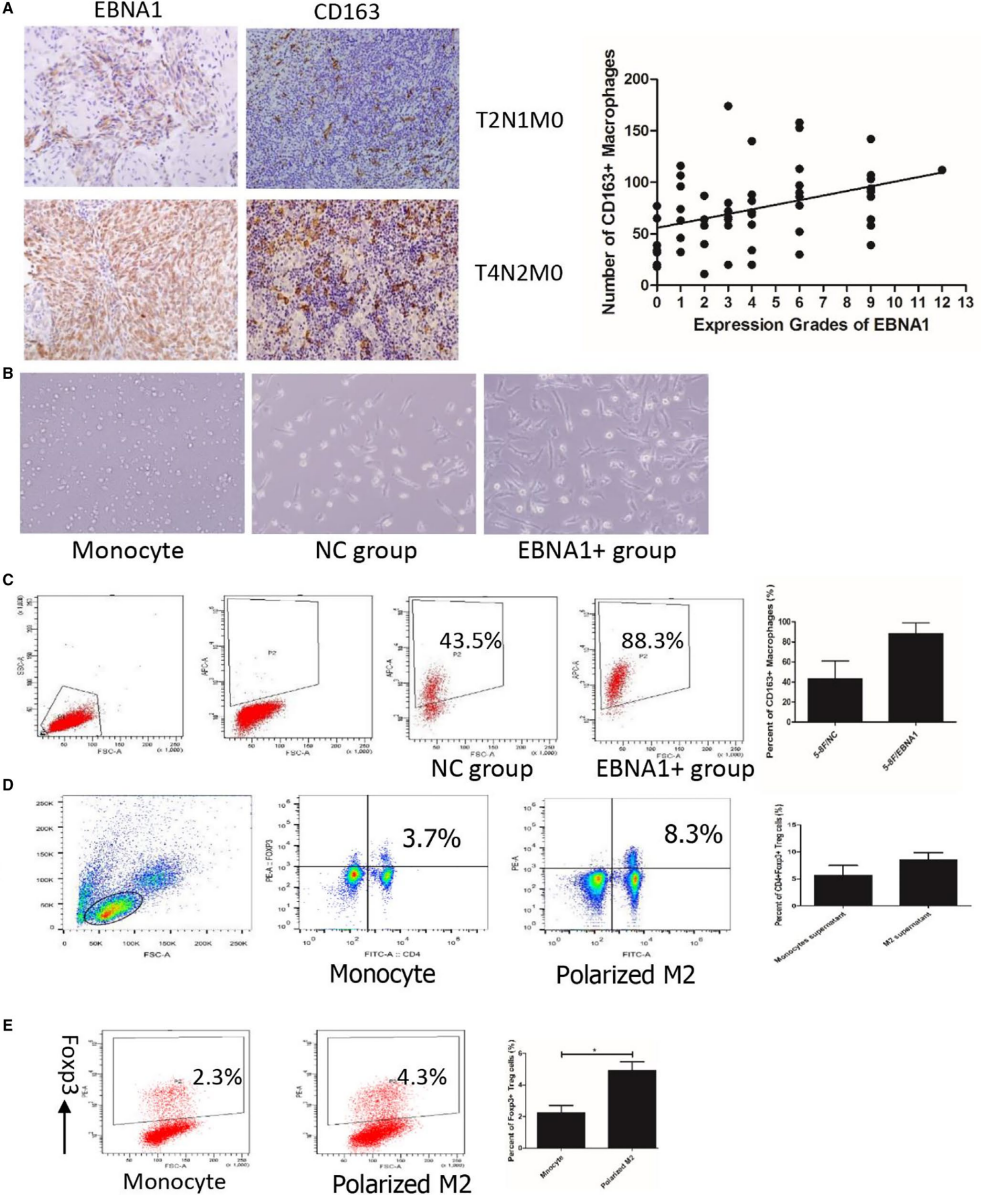
Polarized‐M2 macrophage by EBNA1 converted naïve T cell into Treg. The infiltrated M2 macrophage was positively correlated with EBNA1 expression (A). After cocultured with EBNA1+ cells, the cell got bigger, irregular, and with long lamellipodium (B). EBNA+ cells polarized monocyte into M2 macrophages (C). The percentage of CD4+Foxp3+ Treg in the polarized‐M2 macrophage group was significantly increased (D). Foxp3+ Treg was significantly increased in cocultured with polarized‐M2 macrophage compared with cocultured with monocyte (Figure 5E)

Our previous study proved that infiltrated CD163+ macrophages positively related with infiltrated Foxp3+ Tregs in NPC.^17^ In this study, we chose 50 NPC tissues to analyze the relationship of M2 macrophage and EBNA1 expression. M2 macrophages were mainly distributed in the vicinity of tumors islets. The infiltrated M2 macrophages were positively correlated with EBNA1 expression (Figure 5A).

Freshly isolated human monocytes were small. After cocultured with EBNA1+ cells, the cell got bigger, irregular, and with long lamellipodium (Figure 5B). The percentage of CD163+ M2 macrophages which cocultured with EBNA+ cells (82.7 ± 10.6%) was higher than those cocultured with control (45.1 ± 13.8%) (Figure 5C). EBNA+ cells polarized monocytes into M2 macrophages.

Polarized‐M2 macrophage or monocyte was cocultured with human PBMC for 6 days. The percentage of CD4+Foxp3+ Tregs (8.0 ± 1.1%), which cocultured with polarized‐M2 macrophages group was significantly increased compared with cocultured with monocytes group (3.6 ± 0.8%) (Figure 5D). Then isolated CD4+CD25‐naïve T cells were cocultured with polarized‐M2 macrophages or monocytes. Percentage of Foxp3+ Tregs was higher in cocultured with polarized‐M2 macrophages compared with cocultured with monocytes (Figure 5E).

Animal study revealed xenografts from 5‐8F/EBNA1 was bigger than from 5‐8F/NC (Figure S3A). There were more infiltrated CD4+Foxp3+ Tregs in 5‐8F/EBNA1 xenografts compared with 5‐8F/NC (Figure S3B).

**FIGURE 6:**
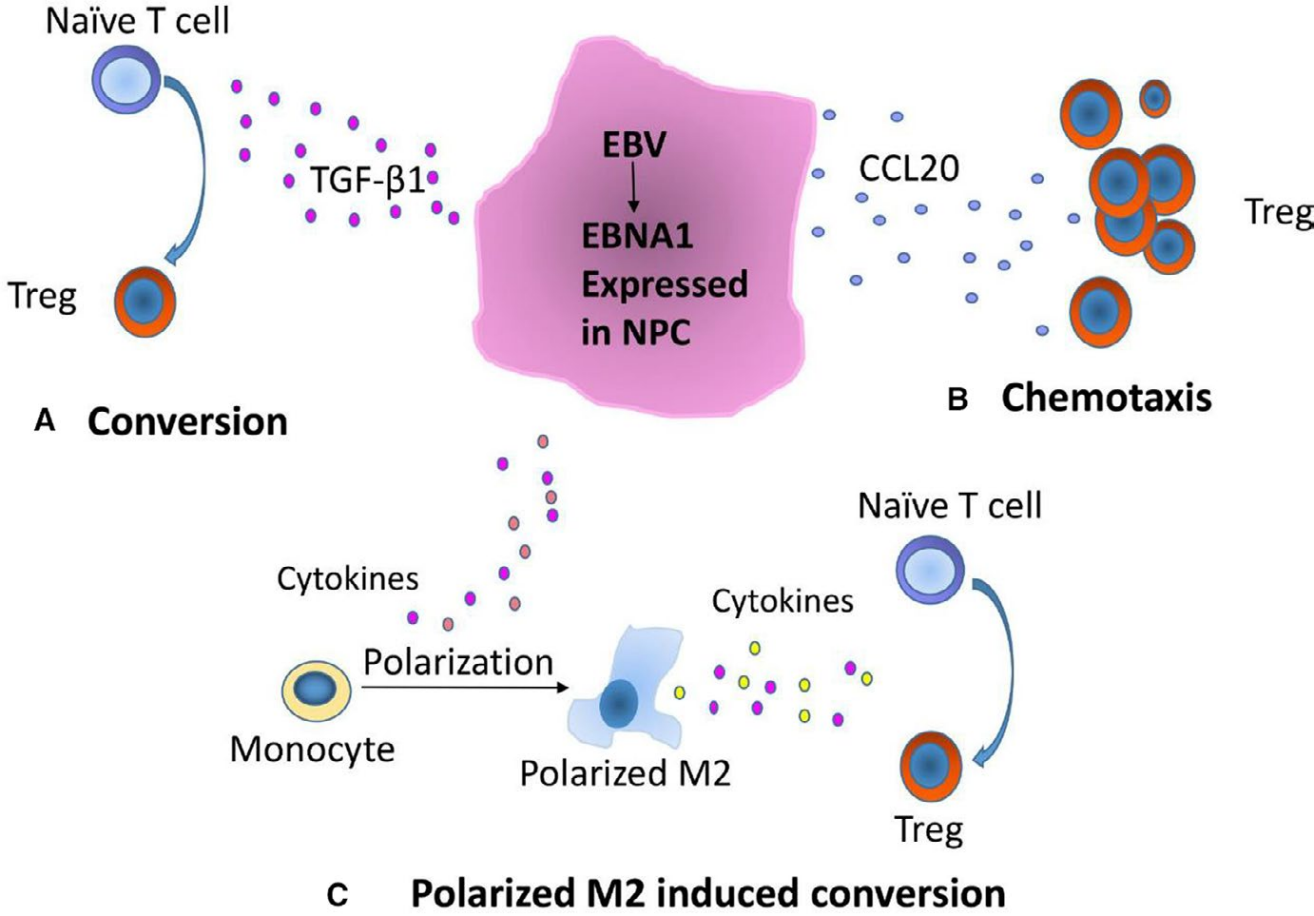
Mechanisms of EBNA1 favor accumulation of Tregs in NPC through: (A) upregulated TGF‐β1 converted naïve T cell into Treg; (B) upregulated CCL20 increased Treg migration; (C) polarized‐M2 macrophage converted naïve T cell into Tregs

In conclusion, mechanisms of EBNA1 favor accumulation of Tregs in NPC through: (a) upregulated TGF‐β1 converted naïve T cell into Tregs; (b) upregulated CCL20 increased Tregs migration; and (c) polarized‐M2 macrophage converted naïve T cell into Tregs (Figure 6).

## 4 DISCUSSION

Although EBV has been conformed could recruit Tregs in other EBV‐associated tumors, the mechanism of Tregs assembled in NPC remains unclear. Our results suggest that EBNA1 favors Tregs recruitment in NPC. We proved the mechanism of EBNA1‐induced accumulation of Tregs through induced naïve T cell conversion into Tregs via upregulated TGF‐β1, upregulated CCL20 which can induce EBV‐directed migration, and polarized‐M2 macrophage convert naïve T into Tregs.

As an EBV‐associated tumor, the characteristic of NPC is a large number of lymphocyte infiltration. Our previous study showed that the number of tumor infiltrating CD8+ CTLs was associated with worse OS in NPC.^17^ This result is in accordance with previous research that EBV‐infected NPC patients are still in a state of immune escape.^18^ EBV viral proteins expressed in these tumors such as EBNA1, LMP1, and LMP2 are immunogenic, yet antiviral immunity cannot eradicate these viral‐associated tumors. It was reported that CD8+ T cells specific for EBNA1 were suppressed in NPC,^1^ as well as LMP2‐specific CD8+ T cells.^2^ In this study, we tested the expression of EBNA1 in NPC tissues, and it was expressed in most of NPC, and stronger stained in late stage (stage III and IV) than early stage (stage I and II). Further analysis revealed expression of EBNA1 had a positive correlation with the number of infiltrated Tregs. This result implied EBNA1 may have the capacity to induce Tregs accumulation in NPC. The accumulation of Tregs in malignant tumors was mainly through conversion, chemotaxis, proliferation, and antiapoptosis.^8^, ^19^, ^20^

Our previous work found overexpressed EBNA1 in NPC cells upregulated TGF‐β1 secreted.^21^ But the mechanism of EBNA1 up‐regulated TGF‐β1 expression remains unclear. We need explore further.

TGF‐β1 converted naïve T cells into Treg was reported in several malignant tumors.^22^ And TGF‐β1‐induced Tregs have the same immune response as thrymus‐derived Tregs.^23^ In this study, we proved the 5‐8F/EBNA1 upregulated secretory protein of TGF‐β1. In vitro, percent of CD4+CD25+Foxp3+ Tregs in PBMC was significantly increased after cocultured with 5‐8F/EBNA1 compared with controls. Furthermore, 5‐8F/EBNA1 cocultured naïve T cells got more Tregs and neutralized TGF‐β1 could weaken this phenomenon. These results indicated EBNA1 induced upregulated TGF‐β1 could convert naïve T cell into Tregs in NPC.

Chemotaxis is another way that Treg accumulated in tumors. It was reported NPC‐derived exosomes carrying CCL20 could recruit Treg.^24^ And the expression of EBNA1 in HL upregulated expression of CCL20 and the migration of Tregs.^9^ It was reported that EBNA1 can activate AP‐1 signaling, and the CCL20 promoter has an AP‐1 binding site.^9^ In our study, we tested expression of nine chemokines in 5‐8F/EBNA1. And the followed test indicated that CCL20 was overexpressed in NPC tissues and 5‐8F/EBNA1 cell line. Moreover, as demonstrated by transwell assay, the enhanced CCL20 caused Treg migration in vitro. In a word, EBNA1 upregulated expression of CCL20 and migration of Treg in NPC.

Our previous study proved that tumor infiltrating M2 macrophages is associated with high expression of EBER in NPC. For further research, we detected the number of M2 macrophages and the expression of EBNA1 in NPC. The result showed M2 macrophages is associated with high expression of EBNA1 in NPC. Blood monocytes could be converted into M2 macrophages by several cytokines such as CSF1, TGF‐β, and IL‐4.^25^ Tumor cells were reported to polarize M2 macrophages in many different cancers, but in NPC this phenomenon has been reported.^26^, ^27^ In this study, we demonstrated that expression of EBNA1 in NPC cell could polarize M2 macrophage. But the mechanisms of polarized‐M2 macrophage need to be further investigated. In the tumor, M2 macrophages were characterized as inhibitors of T‐cell.^28^ Naïve T cells converted into Tregs require TGF‐β and IL‐2, and M2 macrophages and tumor cells are major source of TGF‐β.^29^ In our study, PBMCs were cocultured with polarized‐M2 macrophages, and the percentage of CD4+Foxp3+ Tregs increased compared with control These results indicated polaried‐M2 macrophage could induced naïve T cell converted into Tregs.

In conclusion, mechanisms of EBNA1 favor accumulation of Treg in NPC through: (a) upregulated TGF‐β1 converted naïve T cell into Treg; (b) upregulated CCL20 increased Tregs migration; and (c) polarized‐M2 macrophage converted naïve T cell into Tregs. A deep insight of Tregs accumulation would favor to regulate the balance between NPC and tumor immune escape, thus promoting the future immunotherapy in NPC.

## CONFLICT OF INTEREST

The authors have no competing interests.

## AUTHOR CONTRIBUTIONS

Jie Wang and Xiangping Li conceptualized the study. Patient enrollment was done by Yunfan Luo, Pei Bi, and Juan Lu. Specimen preparation and laboratory assays were done by Fan Wang, Xiong Liu, and Bao Zhang. All the authors contributed to the manuscript and approved the final version.

## Supporting information

Fig S1‐S3

Table S1‐S2

## Data Availability

All of our data are availability.
