# Supplementary material for: Mechanisms of Epstein‐Barr virus nuclear antigen 1 favor Tregs accumulation in nasopharyngeal carcinoma
Source: Cancer Med. 2020 Jun 22;9(15):5598–608. doi: 10.1002/cam4.3213 (PMC7402843; doi:10.1002/cam4.3213)
Supplement: Supplementary file 1 — Fig S1‐S3 [file CAM4-9-5598-s001.pdf]

Figure 1

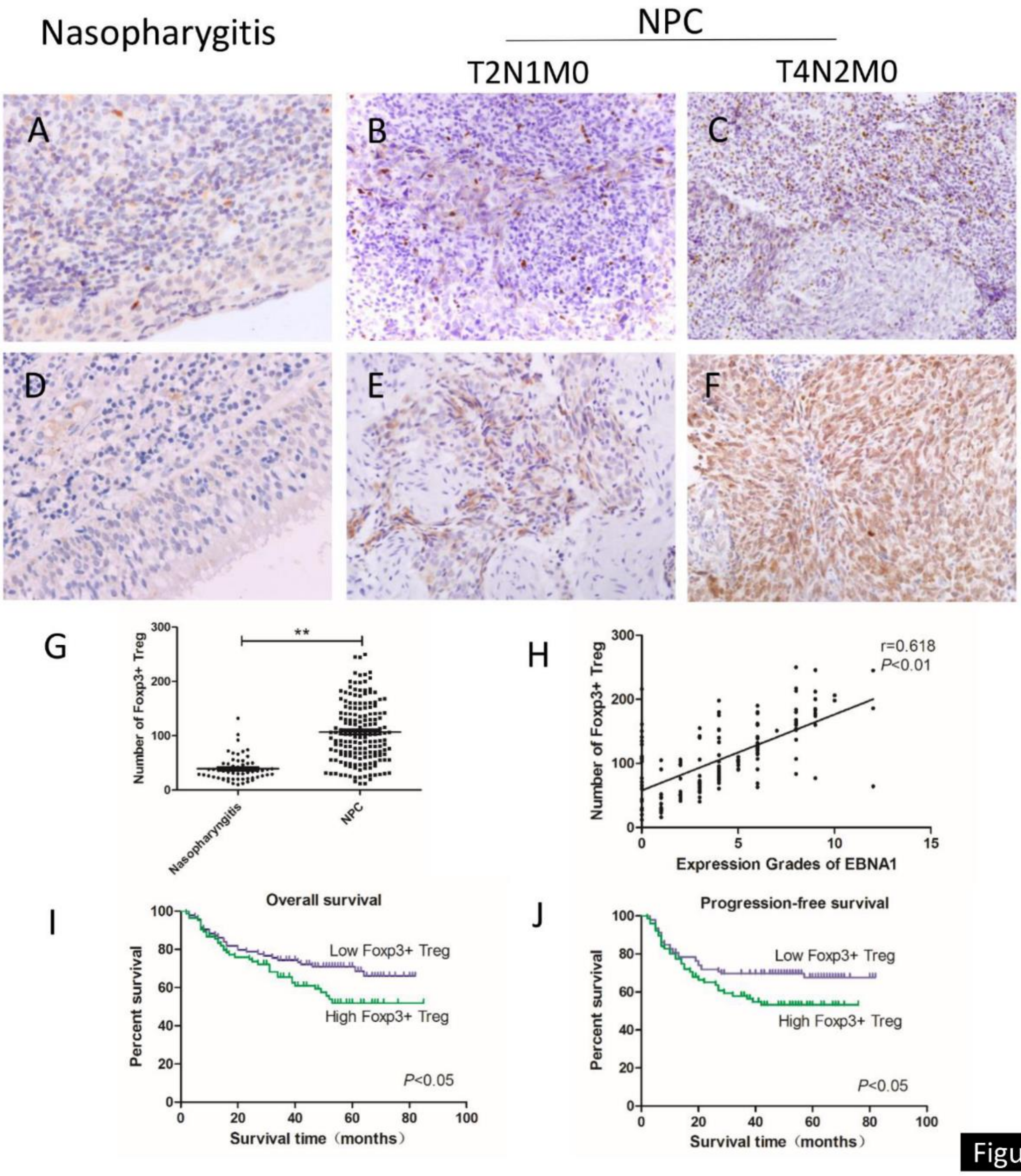

Figure 1

Figure 2

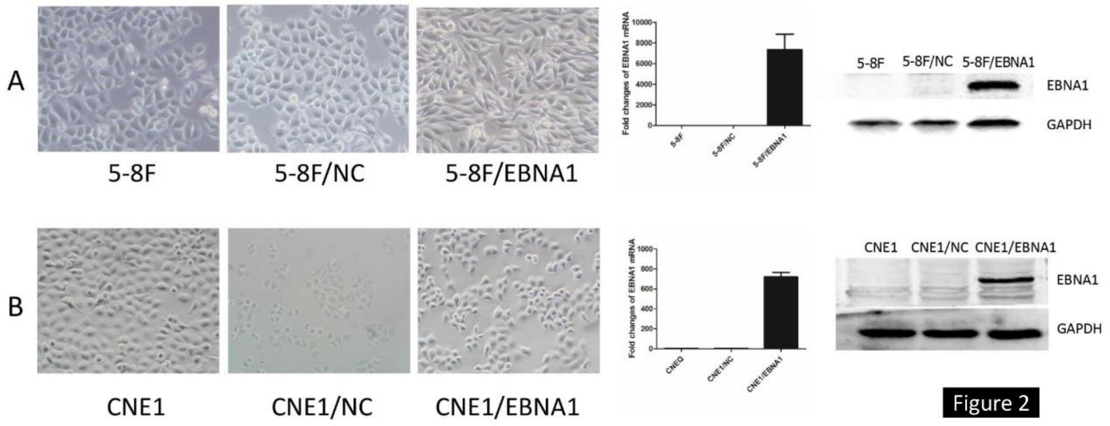

A
5-8F
5-8F/NC
5-8F/EBNA1
Fold changes of EBNA1 mRNA
10000
8000
6000
4000
2000
0
5-8F
5-8F/NC
5-8F/EBNA1
EBNA1
GAPDH
B
CNE1
CNE1/NC
CNE1/EBNA1
Fold changes of EBNA1 mRNA
1000
800
600
400
200
0
CNEQ
CNE1/NC
CNE1/EBNA1
EBNA1
GAPDH
Figure 2

Figure 3

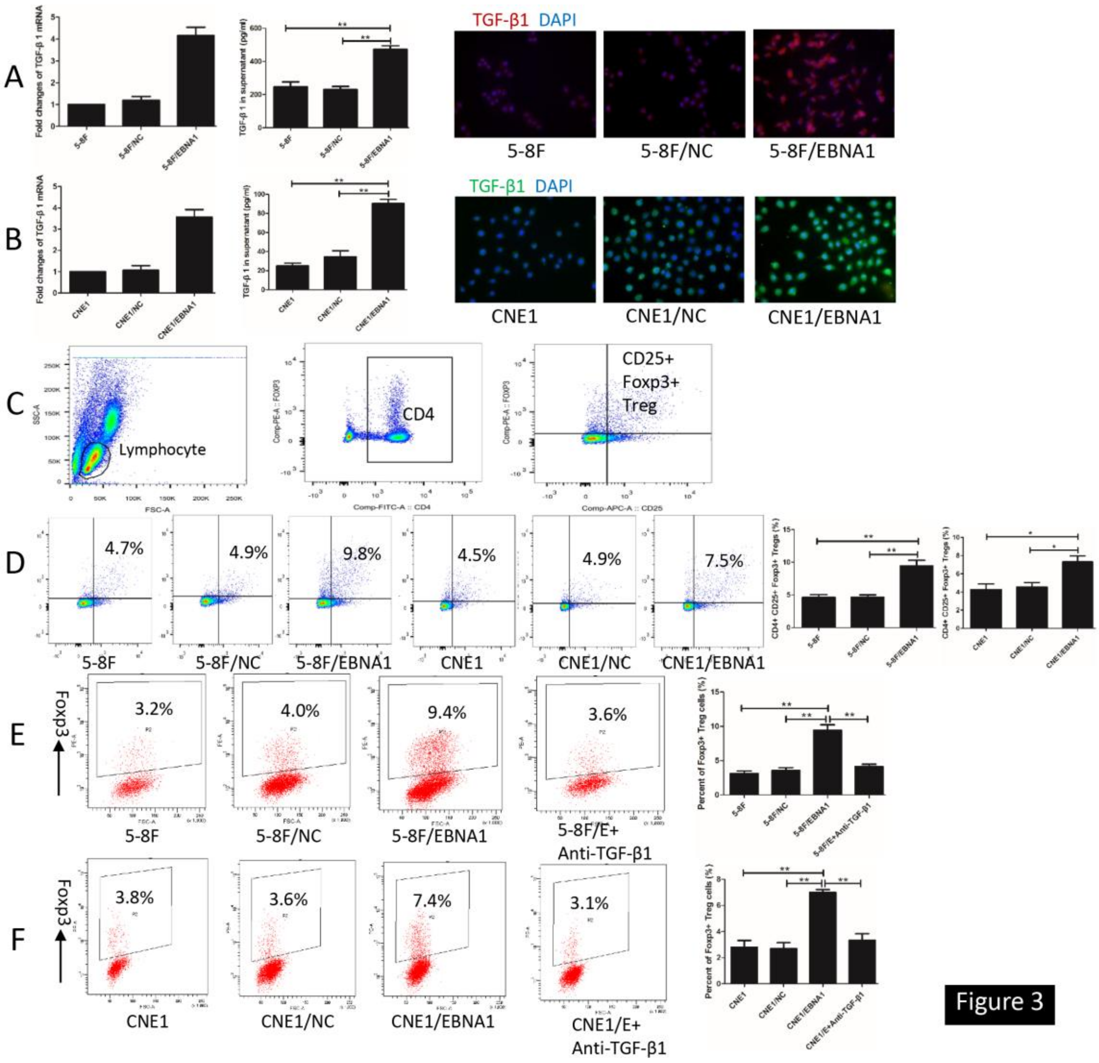
A
B
C
D
E
F
TGF-β1 DAPI
5-8F
5-8F/NC
5-8F/EBNA1
CNE1
CNE1/NC
CNE1/EBNA1
Lymphocyte
CD4
CD25+
Foxp3+
Treg
4.7%
4.9%
9.8%
4.5%
4.9%
7.5%
Foxp3
3.2%
4.0%
9.4%
3.6%
5-8F/E+
Anti-TGF-β1
3.8%
3.6%
7.4%
3.1%
CNE1/E+
Anti-TGF-β1
Figure 3

Figure 4

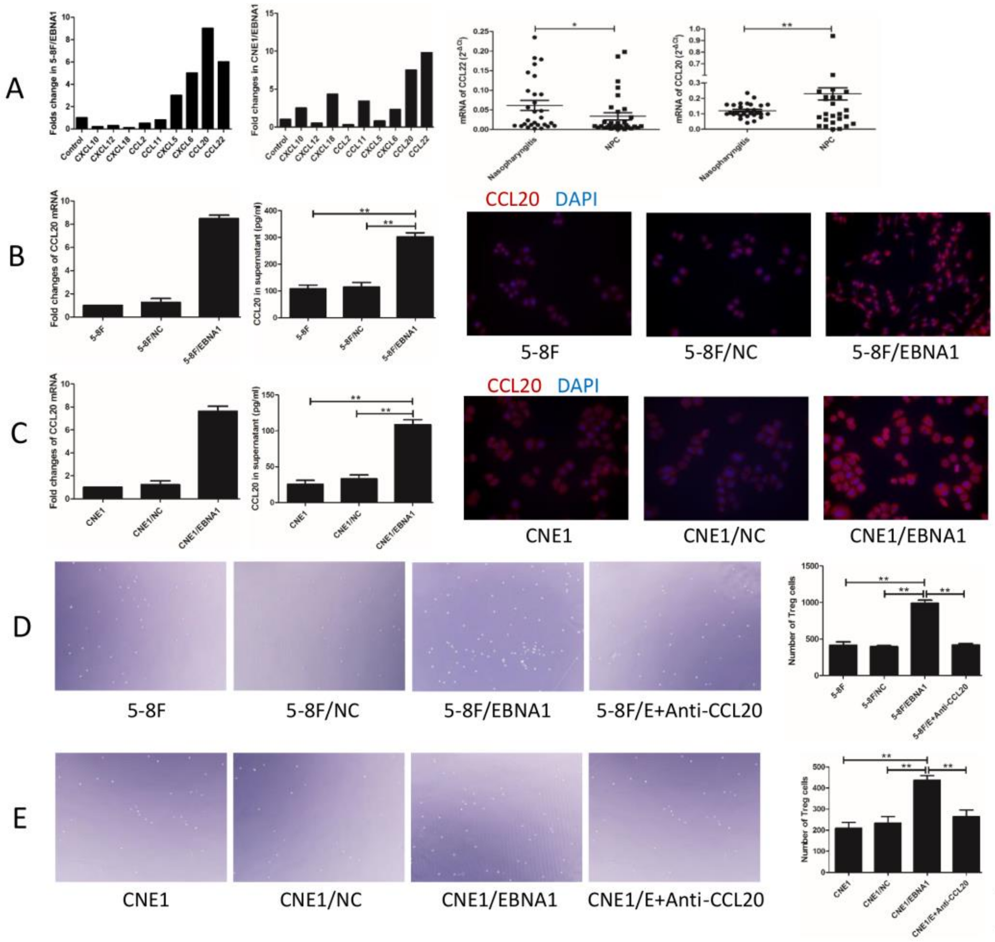

Figure 4

Figure 5

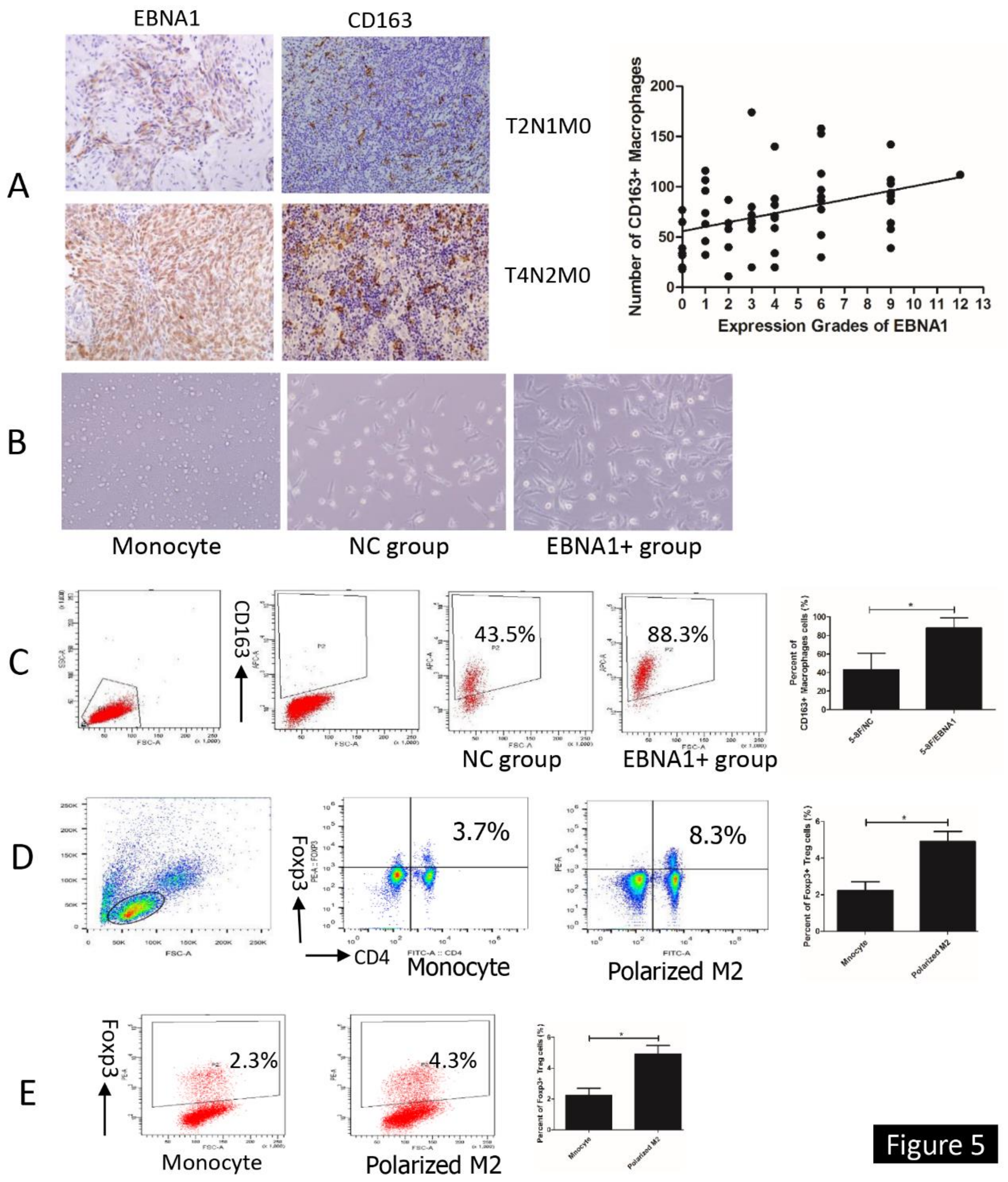
EBNA1
CD163
T2N1M0
T4N2M0
A
Number of CD163+ Macrophages
Expression Grades of EBNA1
B
Monocyte
NC group
EBNA1+ group
C
CD163
43.5%
88.3%
NC group
EBNA1+ group
Percent of CD163+ Macrophages cells (%)
5-8F/NC
5-8F/EBNA1
D
Foxp3
CD4
3.7%
8.3%
Monocyte
Polarized M2
Percent of Foxp3+ Treg cells (%)
Mnocyte
Polarized M2
E
Foxp3
2.3%
4.3%
Monocyte
Polarized M2
Percent of Foxp3+ Treg cells (%)
Mnocyte
Polarized M2
Figure 5

Figure 6

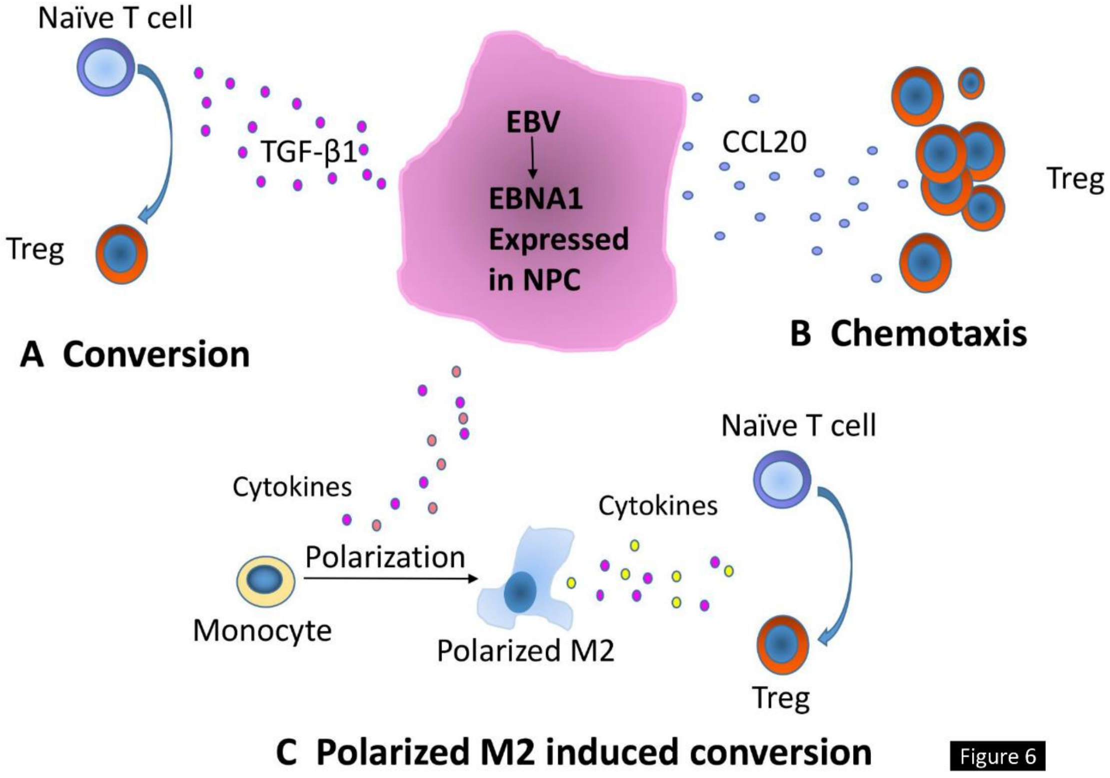

Figure 6

Supplemental Figure 1

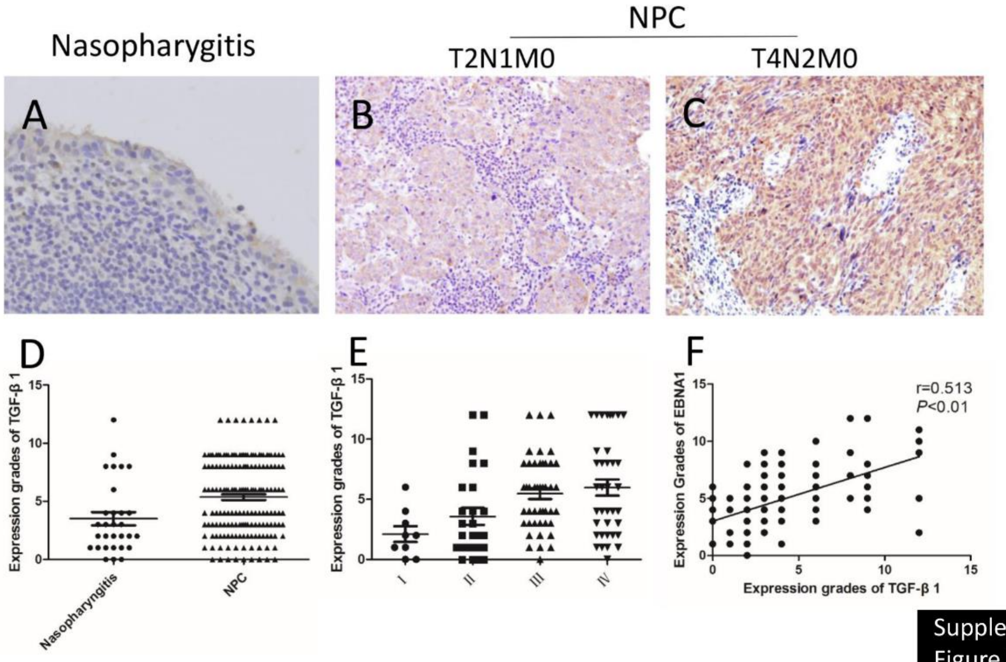

Supplemental Figure 1

Supplemental Figure 2

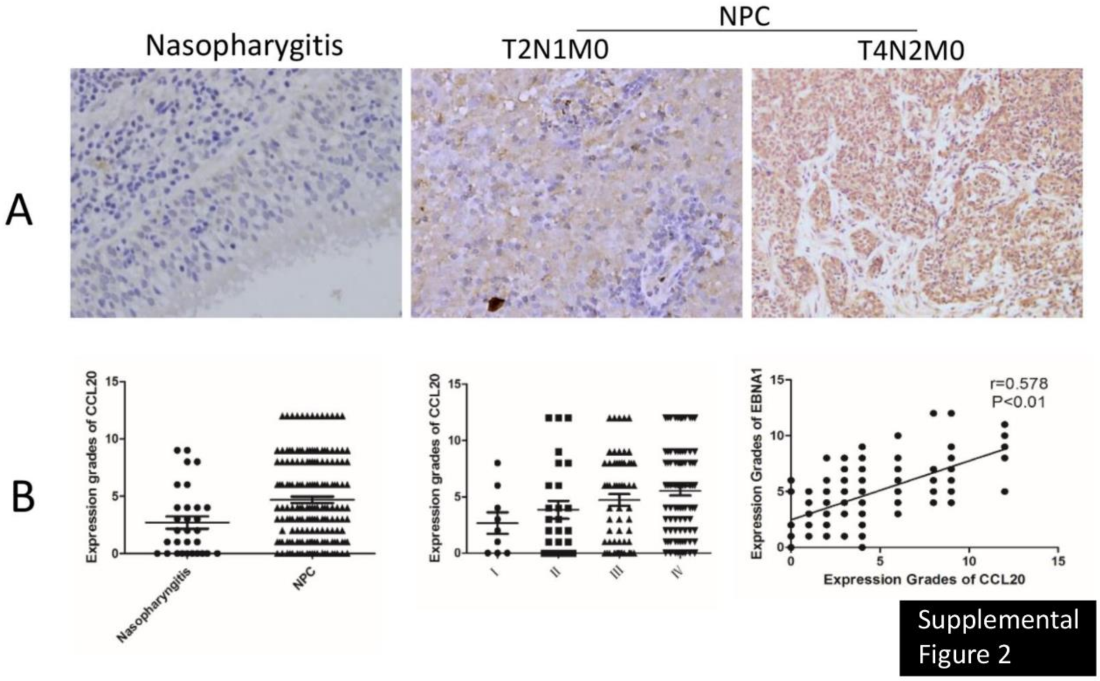

Supplemental Figure 3

A

B

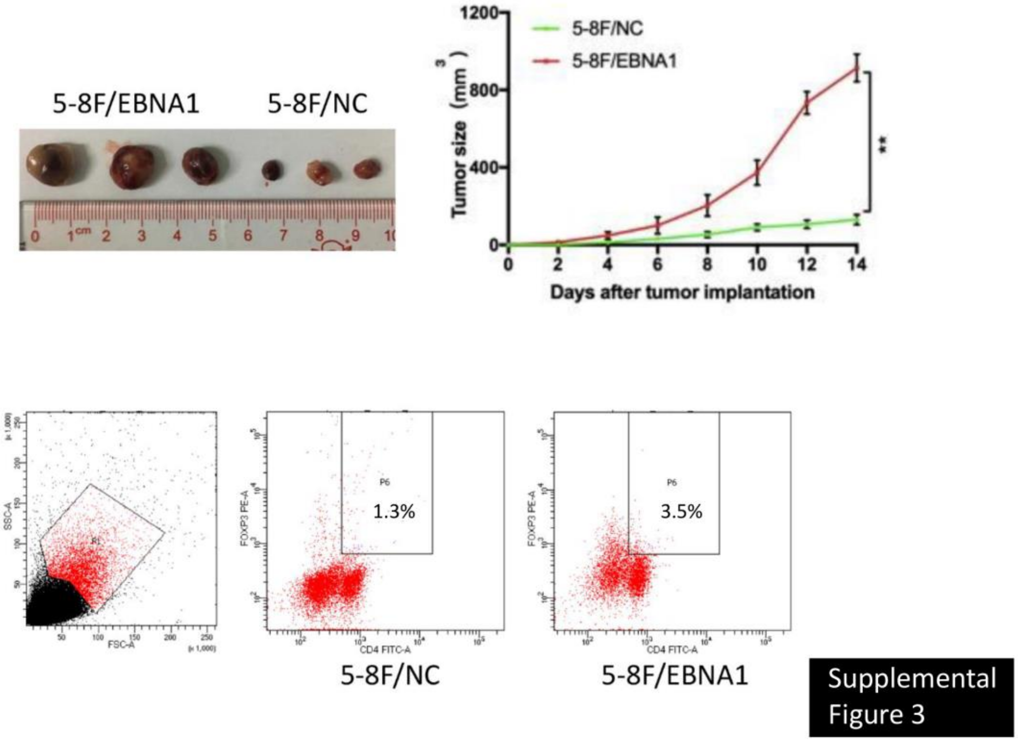

Supplemental Figure 3
