# Supplementary material for: Mechanisms of Epstein‐Barr virus nuclear antigen 1 favor Tregs accumulation in nasopharyngeal carcinoma
Source: Cancer Med. 2020 Jun 22;9(15):5598–608. doi: 10.1002/cam4.3213 (PMC7402843; doi:10.1002/cam4.3213)
Supplement: Supplementary file 2 — Table S1‐S2 [file CAM4-9-5598-s002.docx]

Supplemental Table 1 Univariate Cox analysis of Foxp3+ Tregs and Clinicopathological characteristics in NPC patients.

| Clinicopathological characteristics | Overall survival | | | Progression free survival | | |
| --- | --- | --- | --- | --- | --- | --- |
|  | HR | 95%CI | P | HR | 95%CI | P |
| Gender  (Male/Female) | 0.716 | 0.398-1.292 | 0.271 | 0.552 | 0.303-1.003 | 0.053 |
| Age  (<45/≥45) | 1.478 | 0.923-2.343 | 0.123 | 1.376 | 0.844-2.084 | 0.223 |
| Cigratte Smoking (Yes/No) | 1.258 | 0.845-2.167 | 0.209 | 1.467 | 0.934-2.334 | 0.092 |
| Alcohol Intake (Yes/No) | 1.120 | 0.647-1.876 | 0.765 | 1.234 | 0.765-2.023 | 0.396 |
| T Status  （1+2/3+4) | 2.034 | 1.178-3.488 | **0.015** | 1.732 | 1.023-2.518 | **0.035** |
| N Status  (0+1/2+3) | 2.756 | 1.645-4.576 | **<0.001** | 3.156 | 1.932-5,234 | **<0.001** |
| M Status  (0/1) | 4.987 | 2.345-9.568 | **<0.001** | 4.987 | 2.634-9.508 | **<0.001** |
| Clinical stage  (Ⅰ+Ⅱ/Ⅲ+Ⅳ) | 4.578 | 1.620-12.345 | **0.002** | 5.145 | 1.872-13.176 | **0.001** |
| Foxp3+ Tregs (Low/High) | 1.935 | 1.192-3.195 | **0.024** | 0.962 | 0.382-0.945 | **0.029** |

Supplemental Table 2 Multivariate Cox analysis of Foxp3+ Tregs and Clinicopathological characteristics in NPC patients.

| Clinicopathological characteristics | Overall survival | | | Progression free survival | | |
| --- | --- | --- | --- | --- | --- | --- |
|  | HR | 95%CI | P | HR | 95%CI | P |
| T Status  (1+2/3+4) | 2.871 | 1.574-4.354 | **0.001** | 1.952 | 1.343-2.987 | **0.045** |
| N Status  (0+1/2+3) | 3.347 | 1.968-5.078 | **<0.001** | 4.753 | 2.459-7.348 | **<0.001** |
| M Status  (0/1) | 5.042 | 3.164-8.582 | **<0.001** | 5.168 | 2.673-10.264 | **<0.001** |
| Clinical stage  (Ⅰ+Ⅱ/Ⅲ+Ⅳ) | 4.205 | 2.125-9.654 | **0.004** | 4.964 | 2.822-10.146 | **<0.001** |
| Foxp3+ Tregs  (Low/High) | 1.714 | 1.042-2.843 | **0.034** | 1.543 | 0.806-2.078 | 0.295 |
